# Barriers and facilitators to effective cervical cancer screening in Belize: a qualitative analysis

**DOI:** 10.1007/s10552-023-01703-0

**Published:** 2023-05-11

**Authors:** Avni Mittal, Shane S. Neibart, Abha Kulkarni, Taylor Anderson, Shawna V. Hudson, Natalia Largaespada Beer, Mark H. Einstein, Racquel E. Kohler

**Affiliations:** 1 Department of Obstetrics and Reproductive Health and Gynecology, Rutgers New Jersey Medical School, Newark, NJ USA; 2 Rutgers Robert Wood Johnson Medical School, New Brunswick, NJ USA; 3 University of Queensland Ochsner Clinical School, Brisbane, QLD Australia; 4 Department of Cancer Prevention and Control, Rutgers Cancer Institute of New Jersey, New Brunswick, NJ USA; 5 Ministry of Health, Belmopan, Belize; 6 Department of Health Behavior, Society and Policy, Rutgers School of Public Health , Piscataway, NJ USA

**Keywords:** Cervical cancer, Prevention, Qualitative, Belize

## Abstract

**Purpose:**

Belize has among the highest cervical cancer incidence and mortality rates of Latin American and Caribbean countries. This study evaluates the perspectives of key stakeholders for cervical cancer screening in Belize and identifies the barriers and facilitators for providing equitable access to prevention services.

**Methods:**

Semi-structured interviews discussing cervical cancer screening were conducted with key stakeholders across the six districts of Belize in 2018. Interviews were transcribed, coded, and analyzed thematically; themes were organized by levels of the social-ecological model.

**Results:**

We conducted 47 interviews with health care providers (45%), administrators (17%), government officials (25%), and other stakeholders (13%). Majority (78%) of interviews were from the public sector. Perceived barriers to cervical cancer screening were identified across multiple levels: (1) Individual Patient: potential delays in Pap smear results and fear of a cancer diagnosis; (2) Provider: competing clinician responsibilities; (3) Organizational: insufficient space and training; (4) Community: reduced accessibility in rural areas; and (5) Policy: equipment and staffing budget limitations. The main facilitators we identified included the following: (1) at the Community level: resource-sharing between public and private sectors and dedicated rural outreach personnel; (2) at the Policy level: free public screening services and the establishment of population-based screening.

**Conclusion:**

Despite free, publicly available cervical cancer screening in Belize, complex barriers affect access and completion of management when abnormal screening tests are identified. Provider workload, education outreach, and additional funding for training and facilities are potential areas for strengthening this program and increasing detection and management for cervical cancer control.

**Supplementary Information:**

The online version contains supplementary material available at 10.1007/s10552-023-01703-0.

## Introduction

Cervical cancer is the 3rd leading cause of cancer and 4th leading cause of cancer-related death in women in Latin America and the Caribbean (LAC) countries [1]. While cervical cancer prevention programs have reduced the disease burden in high-income countries, low- and middle-income countries shoulder nearly 87% of global cervical cancer deaths [1]. The high burden of cervical cancer in LAC countries epitomizes the global inequity of this preventable disease [2, 3]. To combat this public health issue, in 2018, the WHO announced an initiative to eliminate cervical cancer, setting country-wide vaccination, screening, and treatment targets to achieve by 2030. Notably, 70% of women should be screened using a high-performance test by age the age of 35 and again by 45 [4].

Belize, a country of just over 400,000 citizens on the eastern coast of Central America, is not exempt from challenges in cervical cancer management [5]. The 2020 estimates of cervical cancer incidence and mortality (19.1 and 14.8 cases per 100,000, respectively) are among the highest in LAC (14.9 and 7.6 cases per 100,000 population) [1]. The burden of cervical cancer in Belize not only strains the healthcare system, but also impacts the overall economy through the indirect productivity losses from women in the workforce, and due to mortality, care of their children by the family and community [6, 7]. Addressing premature loss of life from cervical cancer has ethical and economic appeal.

The Ministry of Health (MOH) in Belize has implemented policies aiming to increase access to cervical cancer prevention and treatment resources. In 2016, Belize launched a national human papillomavirus (HPV) vaccination program for adolescents in collaboration with the Ministry of Education [8, 9]. At the same time, the national cervical cancer prevention effort was strengthened through implementing visual inspection with acetic acid (VIA) screening protocols and resources in the public sector [10]. The efforts to scale cancer prevention and control services have resulted in an estimated 49% screening coverage of women ages 35–49, still shy of Belize’s goal of screening 80% of women. These programs to improve cervical cancer care were bolstered by the opening of Belize’s first medical oncology clinic in the public sector in 2018 [11, 12].

The real-world impact and implementation of expanded services in Belize have not been evaluated explicitly. This study is part of a larger mixed-methods assessment of cervical cancer prevention and control in Belize [10]. In our prior study, we documented limited personnel participating in outreach to rural communities, geographic barriers to accessing services, and stresses on the pathology infrastructure that impact the diagnosis and screening of cervical cancer in Belize. In this qualitative component, we analyzed interview data from key stakeholders (e.g., health care providers, policy-makers, and patient advocates) to identify the barriers and facilitators of cervical cancer screening services to inform future national and local district policy development.

## Methods

### Study design

We collaborated with MOH officials involved in implementing the cervical cancer screening program to generate a list of key stakeholder categories and contacts at the national level and across each of the six districts. We also employed snowball sampling to recruit additional participants within the districts and stakeholder categories to ensure a variety of perspectives at a grass roots level were included. We aimed to recruit participants involved in various aspects of screening: patient advocates, outreach educators, clinicians conducting screening, laboratory and technology staff processing the results, and administrators overseeing services and facilities. To protect patient confidentiality and abide by privacy standards in Belize, individual patients were not interviewed. This study was approved by the Institutional Review Board at the Rutgers New Jersey Medical School and by the Director of Health Services at the MOH in Belize.

### Data collection

The semi-structured interview (SSI) guide (Supplement) was developed in consultation with MOH officials and pilot tested with experts at the MOH before deployment. Questions were largely based on aspects of the WHO’s health system building blocks: human resources, infrastructure, equipment, and policies that facilitate or impede access to cervical cancer screening services. All interviews were conducted in person at the interviewees workplace, in English, in private, and audio recorded by a single doctorate-candidate, male, researcher (SN) in June 2018. At the start of each interview, the interviewer (SN) introduced his background, described the aforementioned objectives of the study, and ensured confidentiality. We mainly conducted individual SSIs (64%); however, dyad interviews were conducted when two individuals from the same facility and of the same role wanted to participate. All interview participants provided informed consent for data collection prior to the discussion. Limited field notes were collected during interviews, and all interviews were transcribed after all interviews were conducted.

### Thematic framework: the social-ecological model

Health care delivery is a complex process with multifaceted barriers to access. McLeroy’s social-ecological model (SEM) supports the idea that public health problems, such as access to cervical cancer screening, are the result of both individual and social environmental factors. This model posits that “behavior is viewed as being determined by intrapersonal, interpersonal, institutional, community and public policy factors which can be used to "identify strategies for health promotion programming” We adapted McLeroy’s social-ecological model to help understand the multiple factors that intersect and effect screening as our framework for organizing concepts and themes [13].

### Analysis

Two co-authors (AM, SN) read and reviewed all transcripts, working with the team to develop the analytic approach to thematic content analysis. We developed our coding framework with initial codes such as pap-delay, local service availability, and resources, stemming from the interview guide topics and additional codes being added as they emerged from the data using the social-ecological model as a framework. Using the final codebook, two researchers (AM and SN) independently coded 10 interview transcripts using NVivo version 12 (QSR International, Doncaster, Australia) to compare the application of codes and resolve discrepancies as needed. Inter-coder reliability was calculated using Cohen’s Kappa coefficient, A 0–1 scale where a coefficient of 1 means a perfect agreement between the coders [14]. The calculated kappa after the first 10 interviews was 0.94 indicating a nearly perfect agreement between AM and SN. All further coding was completed by the lead author (AM). The team reviewed coding reports to discuss findings and themes. This study followed the Consolidated Criteria for Reporting Qualitative Research (COREQ) guidelines [15].

## Results

### Sample characteristics

We conducted 47 interviews, 30 individual and 17 dyad, with a total of 64 participants (Table 1). There were no individuals approached who refused participation. While all regions were represented in the analysis, stakeholders affiliated at the national level represented 40% of participants. A majority (78%) of participants worked in the public sector. Over half of the stakeholders were directly involved in health care delivery (57%). Although no patients were interviewed, 8% of interviews were conducted with members of patient advocacy groups from four out of six districts.

**Table 1:** Interview characteristics

Characteristic *n* = 47	No. (%)
**Number of participants per interview**	
1	30 (63.8)
> = 2	17 (36.2)
**Sector**	
Public	39 (83.0)
Private	8 (17.0)
Region*	
National	19 (40.4)
Northern	8 (17.0)
Central	9 (19.2)
Western	5 (10.6)
Southern	6 (12.8)
**Stakeholder type**	
Administration	8 (17.0)
Advocacy	4 (8.5)
Supportive	3 (6.4)
Services**	27 (57.5)
Healthcare Team^+^	5 (10.6)
Ministry of health/national offices	

*N* = 47, subsample size defined where relevant

*Regions presented instead of districts to protect anonymity; Northern Region (Corozal, Orange Walk Districts), Central Region (Belize District), Western Region (Cayo District), Southern Region (Stann Creek, Toledo Districts)

**Supportive services include, facility engineering support, information technology, and supply chain

^+^Healthcare team includes medical doctors specializing in oncology, gynecology, pediatrics, primary care, palliative care and pathology, RNs, and technicians

**Fig. 1:**
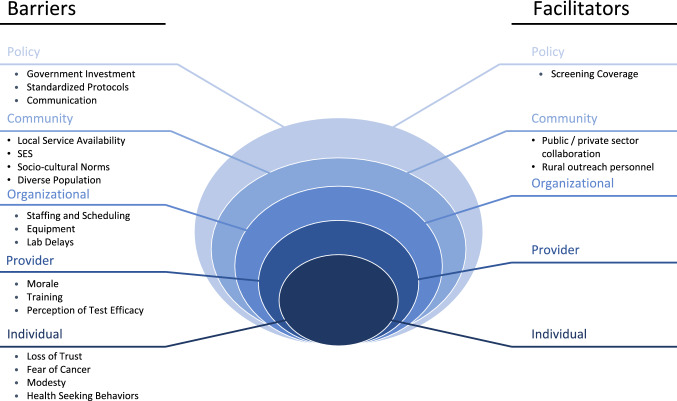
Barriers and facilitators of cervical cancer screening in Belize using the socio-ecological model

We identified barriers and facilitators to screening for cervical cancer in Belize across multiple social-ecological levels as illustrated in Fig. 1. Participants were most concerned with barriers at the organizational and community levels: training, staffing, morale, equipment, and local service ability. Key quotations from our interviews which best embody the barriers to screening across multiple levels of influence can be found in Table 2.

**Table 2:** Key thematic barriers across multiple levels of influence

SEM level	Theme	Key quotation (Interview No.)
**Individual Patient**	Loss of trust	“We counsel them, and they come in two months' time… and they ask for their results and it’s not here yet, so they are kind of discouraged to keep on doing the pap smear” Healthcare Provider (4)
	Fear of cancer	“The problem [with screening] was that, a patient with cervical cancer in a late stage needs any support of palliative treatment. Most of the treatment is only offered by our tertiary and most of the time it’s denied. In response we get this, that she will die or he will die. Why do it?” Public Administration (9)
**Provider**	Provider training	“So the training that they gave us was like a one week training. I feel that we might need more, …What I noticed though is that sometimes I do question what I see because there's certain patients that you're not really sure of them…I’ll just do a Pap smear. So, in that case maybe like a little bit more training would help maybe a second training. Healthcare Provider (10)
	Perception of VIA Efficacy	“When you talk about access, I like to say true Access. VIA [improves] access, cost and human resources because the nurses are already at these centers, they nurses are already doing the work, [and] we don't have the capacity a central lab [to process cytology samples] … It’s what works!” Advocacy Group (1)
**Health Care System**	Laboratory delays	“So we advocate for Pap smear, but then behind your mind, you know that this woman is not getting results right away. So that discourages women of doing their Pap smear and that's where the VIA would come in more handy you know” Healthcare Provider (11)
	Workload and scheduling	“It’s a lot of work for one nurse, because the VIA is not only a patient. No, you have to do an educational counselling first. So, that’s a process in doing a VIA which can end up with a LEEP or a cryotherapy…” Healthcare Provider (33)
	Equipment and facilities	“We need more space, office space for nurses. If we’re going to train more nurses we need more space for them to be able to deliver the service.” Private Administrator (20)
**Community**	Local service availability	“Most villages have access to our institution; the problem comes with the farthest one… they have to cross two ferries and the road is a dirt road which with rain is impossible to get out” Public Sector Administrator (9)
	SES	“Poverty is very high here. Priorities are very disorganized in people’s lives. Health is not a priority for everybody” Public Sector Administrator (6)
	Sociocultural norms	“So, we still have females who need to like actually get consent from the husband or saying that they don't really want to do it unless they speak to their husbands before” Healthcare Provider (30)
	Diverse population	“English is [some Mayans] third language and some of them have difficulty understanding, and even for us, it is difficult for us to translate the information form English (especially medical terms) to Kekchi or Mopan Mayan. So they might understand 75% of our message” Advocacy Group (3)
**Policy**	Protocol	“The thing is here we have to standardize the method; we haven’t standardized the method as such for screening. So, we know that according to WHO guidelines all are valid. So, I think our only issue would be standardize at the national level what do we want, what would be more convenient for the country” Healthcare Provider (14)

At the individual patient level, several barriers to accessing care emerged including ability to pay, loss of trust in the public sector, cultural modesty, fear of cancer, and health literacy. The MOH provides screening with no out-of-pocket payment, which participants noted was a facilitator for many patients to access screening. However, participants noted that the private sector was better equipped to deliver accurate results in a timely manner. Therefore, patients using the public system faced long turnaround times, missing results, and hidden costs. For example, participants noted that although screening required no payment, “if they would like the result to come in earlier…they would [have to] pay for” processing the sample in the private sector. These barriers contributed to patient frustrations and mistrust in the reliability of the public screening system. Multiple physicians explained that their patients were “kind of discouraged to keep on doing the Pap smear” and that they “don’t come back […] because they haven’t gotten the results yet.” Participants also observed low knowledge about the importance of screening, noting that many women presented for Pap smears only after experiencing vaginal bleeding, often resulting in late-stage diagnoses of cervical cancer. Poor health-seeking behaviors and limited reach of current education efforts in underserved areas were also noted.

*“But then the challenge is to get to that group who has never got a screening, who has never been consistent in the screening...” (Public Sector Healthcare Provider, 33)*

Providers also recalled their patients saying, “I am scared to find out I had something,” referring to their fear of dysplasia or a pre-cancer diagnosis. Moreover, providers discussed how the lack of accessible tertiary care made patients feel that they “will die anyway…why do it?” when asked about screening. Many health care providers indicated that personal beliefs around modesty kept some women from getting screened, noting “they feel reluctant [to have the] male doctors doing the procedure.”

On the provider level, salient themes emerged around communication and language, VIA and cytology skills, and workload. Providers discussed the benefits of VIA screening, namely that increased access to screening and same-day treatment helped patients who may otherwise be lost to follow-up, noting that “the beauty of it is that the patient gets the result immediately.” A participant further discussed VIA’s role:

*VIA [improves] access, cost and human resources because the nurses are already at these centers, the nurses are already doing the work, [and] we don’t have the capacity [to reliably process cytology samples]” (Advocacy Group, 1)*

Although participants acknowledged the positives of VIA as a screening method, they also expressed concerns about the subjective nature of VIA and lack of quality assurance procedures.

*Because the VIA depends a lot on the personnel – training – and the equipment that we use. I feel more comfortable with the Pap smear. If I see an abnormality and I am not sure, I do a Pap smear for confirmation.” (Healthcare Provider, 7)*

Some providers preferred cytology, but laboratory delays of results and inadequate Pap results due to poor sampling or poor processing technique decreased their confidence in cytology as a screening modality. They were concerned about insufficient training in both screening modalities because “you won’t get good results if you don’t know how to take the sample well.”

Staffing shortages caused issues at both the provider and organizational level as nurses and community workers felt overworked, leading to low staff morale and limited screening service availability. One participant noted that “patient come in for a Pap on a say Monday, but they are only being done on Thursdays because of space and staff, so patients don’t come back” with another provider agreeing that “it depends on the workload if we can accommodate a walk in.” Participants also noted the lack of professionals trained in cervical cancer screening, with one provider adding that “[more training] would alleviate the workload of the Maternal and Child Health (MCH) nurses” and increase the availability of screening personnel given that screening was one of their many other responsibilities. MCH nurses notably are responsible for vaccination, prenatal care, and other preventative gynecology services, so they understandably feel that “It’s overwhelming to have everything else on top of your shoulder and VIA.” Although the MOH encouraged screening services be readily available each day, participants expressed it was “not possible with the staffing situation.”

*We still need more staff to be trained because right now we’re only … overloading the few we have trained. So, and the bulk of work is on them, so, we need to train more staff or… hire more staff, so, that we can do more prevention. (Administrator, 31)*

Additional barriers at the health care organization level included lack of designated space in facilities and insufficient equipment that reportedly undermined providers’ concerted efforts to increase screening. For example, some of the rooms used for VIA screening were shared with the high-risk pregnancy clinic, and other providers often “would want to borrow [the] room.” Some facilities had to share equipment across districts. Faulty equipment was also a commonly mentioned barrier because “equipments are outdated and they’re constantly breaking down.” Participants felt that the sparse staffing and facilities contributed to cytology result delays and even misplaced samples. For example, one physician explained, “it’s frustrating…that we’ve done her Pap smear 3 times and those three times they’ve lost their results.”

Barriers identified at the community environment level included service accessibility, population socioeconomic status, sociocultural norms, and private sector services. Facilitators included public and private sector collaboration and rural outreach programs. While public screening services are available in each district, geographic barriers persist with participants in rural areas noting that “it’s very difficult [for patients] to reach the center.” Additionally, language barriers between patients and providers, especially in these areas of the country, were a perceived limitation to offering screening services. Advocates reported that patients who lacked financial means were also generally unable to take time off from work to make trips to the clinic or pay for tertiary services should cancer be detected. Regional disparities in socioeconomic position were thought to influence not only travel expenses but also patient priorities and health behaviors. This barrier is further exacerbated for some women in Belize by a culture of *machismo*, where women are, at times, pressured to seek permission from their spouse before receiving the funds for medical interventions. Because men in some communities are not educated on the importance of cervical cancer screening and the fact that early detection of dysplasia is treatable and curable, they would often deem screening as an unnecessary cost leaving woman without the means to afford screening. However, some participants mentioned that they “lean on the private sector,” which facilitates access to screening services by sharing equipment, supplies, and manpower with public facilities. Private facilities, although at a higher cost for services provided, were described as having increased trust and shorter wait times, with patients waiting as little as one week for Pap smear results. Participants reported this increased convenience led to “the culture in Belize [being that] most of the patients try to go into the private facility most of the time if they can afford it.”

At the national policy level, barriers included few policies and protocols to guide providers providing screening and treatment services, unclear communication of priorities and programs, and relaxed enforcement of national mandates. In fact, multiple participants wanted increased government involvement. Some stakeholders reported wanting “to standardize the method” to have clear guidance for how to manage patients after screening and felt that the policies and guidelines currently in place were minimally enforced.

Participants wanted to be involved in the decision-making process as a way to increase buy-in, implementation, and enforcement of policies. One physician was frustrated that “some of the policies … are made by the people in authority without consulting with the people who directly are involved.” Others felt equipment and staffing resources were not allocated well leading to a surplus or shortage at different clinic sites. Despite some perceived shortcomings in the government, participants acknowledged that national programming “allowed them to provide free services for …vulnerable populations that would not be addressed otherwise [such as]… sex workers, … women in brothels…, and young persons… which other programs might overlook.”

## Discussion

Our study is the first to evaluate the perspectives of key stakeholders in the national cervical cancer screening program in Belize. We found that barriers to accessing cervical cancer screening were found at all levels of the SEM whereas facilitators were only found at the community and national levels. Recent policy changes to provide screening at no cost and set population-based screening goals must still overcome barriers to cervical cancer screening that exists across individual, provider, organizational, community, and national policy levels.

On the individual patient level, the most salient theme that emerged was patients’ fear of cancer diagnosis and low health literacy regarding the natural history of cervical cancer and treatable forms if diagnosed early. Some patients believe that having access to screening is not enough—if there are no sustainable services offered if dysplasia or cancer is detected, patients would rather not get screened. Not only does this demonstrate a significant barrier to encouraging screening utilization, but it also exemplifies an education challenge where women might not understand the role of asymptomatic screening and identification and treatment of dysplasia. Several studies in Tanzania focused on understanding patient perspectives on cervical cancer mirrored findings seen in our study. Women in Tanzania thought that screening was meant for after symptoms occurred, and a cancer diagnosis was taboo [16]. Many patients don’t wish to be screened because they feel that if cancer is detected, “there is not much to be done,” which notably was not an issue in the middle and upper class [17]. This fatalistic attitude most likely stems from a lack of treatment options in LMICs. In 2020, Global Oncology Inc patterned with Belize’s tertiary care facility to form the first medical oncology clinic in the Belizean public sector, which provides chemotherapy and palliative care options to patients with cervical cancer [18]. While Belize still has no access to radiation therapy in country, this step toward adequate tertiary care access will hopefully lead to increased patient engagement in screening.

On the provider level, we found that while Belize has invested considerable resources to provide access to cervical cancer prevention services in each district [10], there remains the need for additional investment in training of personnel. For example, a deficit of trained workers has created limited availability of screening in Belize. Similar issues were seen in other LMICs where “stability of a trained workforce in many regions was compromised due to high turnover and lack of consistency/durability of contracts for health care professionals” [19]. Many studies have suggested that community health workers (CHW) be employed to fill this deficit with the WHO suggesting “task shifting” certain roles to lesser trained professionals [20]. O’Donovan et al. warns against using CHW as a “silver bullet solution” and calls on policy-makers for adequate training for secondary prevention services [21]. Especially because of the subjective nature of VIA, task shifting might introduce unforeseen consequences of a lack of quality control that can lead to overtreating benign findings or missing cases of invasive cancer that require tertiary services. Training of new healthcare providers, dedicated to providing the VIA services locally and in outreach activities, could improve realized access to care as seen in studies conducted in India, Peru, Vietnam, and Bangladesh [22, 23]. Similarly, the staff trained in providing cervical cancer screening reported morale declining from pressure to balance additional roles and responsibilities. Offering VIA is labor intensive, and although the service might benefit the national screening program in theory, those offering the service are overworked and experiencing burnout. Similar organization level barriers were seen in Tanzania and Uganda where providers noted similar issues of inadequate staffing for cervical screening, limited trained staff resulting in task overload, and staff disinterest in doing the work creating many missed opportunities for cervical screening among clients [16, 24]. According to a 2018 study done in Belize, the public cervical cancer screening workforce in Belize consists of 75 primary care nurses and physicians—one per 1,076 screening-eligible women [10]. Additionally, although “all districts have at least one screening facility, 50% perform screening services only once per week with colposcopy and LEEP available in only three and four districts, respectively,” further emphasizing the service gap created by the lack of trained staff in Belize [10].

At the organizational and community level, the main theme that emerged centered on the importance of establishing and maintaining dedicated facilities and staff for screening services to increase the local service availability of cervical cancer. Because of the shared spaces, offering screening services needs to be coordinated with the weekly schedule of the clinic, which may limit the possibility of offering service to walk-ins. While most services at the point of care do not need advanced equipment, sustainable delivery of pathology services require regular upkeep of equipment and a reliable supply chain for disposables and reagents. In a 2018 assessment of essential pathology equipment nationwide in Belize, 38.5% were present and functional, 23% were present but nonfunctional, and 38.5% were unavailable. Additionally, 35% of supplies were unavailable at the time of assessment, and 75% were unavailable at least once in the 12 months before assessment [10]. Although many countries such as Costa Rica have seen great decline in cervical cancer incidence and mortality since centralizing their cytology laboratories [25], the same might not be true in Belize. As discussed, Pap smear result delay was found to be an issue at the individual, provider, and organizational levels in Belize. In Zimbabwe, the national Pap smear screening program was stopped due to limitations in manpower and infrastructure. The study suggested that in LMICs, training general healthcare providers has helped in decentralizing VIA screening, further demonstrating how investment in training can improve cervical cancer outcomes in Belize [26]. While the public sector has been working to augment training of staff to deliver cervical cancer screening, several respondents commented on public–private partnerships as a facilitator improving access to services. This perspective is supported by WHO’s *Global Strategy to Accelerate the Elimination of Cervical Cancer as a Public Health Problem* [20]*.* Leveraging provision of services in the private sector and even human resources to educate additional healthcare providers to provide screening could improve access and sustainability. While public–private partnerships may benefit populations in urban areas, it is unclear if services will be accessible to the most at-risk population. Only 44% of healthcare workers participating in screening at the health cancer centers are able to conduct outreach events in the local villages [10]. While the presence of this outreach workforce was cited as a facilitator by some respondents, others suggested that additional investment is needed. The same population of women who may have culturally rooted barriers to access at both the individual and community levels are also living in remote villages, thus further complicating improving their access to services. Geographic inaccessibility remains the central barrier to cervical cancer screening in resource poor districts of Peru [27], a finding which was mirrored in the transportation and geographic barriers to screening seen in more rural districts of Belize such as Toledo. Continuing Investment at the organizational and community level by hiring culturally competent staff for outreach who speak the local dialect could address several barriers for this population.

At the national policy level, the themes that emerged centered around stakeholder desire for coordinated implementation of and investment in policies, protocols, and bureaucracy efforts, to effectuate the national screening program. While Belize does have a national cervical cancer screening program and offers no-cost screening for women who access services in the public sector, some stakeholders reported that they wish they were consulted before implementing new screening programs (e.g., VIA). Peters et al. suggests using the participatory action research method where stakeholders perform their own research to inform decision-making as a method to engage stakeholders, which a team in Uganda had great success in doing [28]. Development and implementation of standardized protocols is needed to ensure that advancements in care reach the entire population. This is especially important as the Ministry of Health prioritizes new programs to advance HPV DNA testing as the primary screening modality in Belize in line with updated WHO guidelines [29]. Another limitation to expanding access to cervical cancer screening is that, due to the bureaucracy, screening services might not be equitably distributed across the population.

Our study had a few notable limitations. First, our study used purposive sampling, visiting clinics that the Belize MOH suggested we visit to conduct our interviews, which increased the risk of researcher bias in our data. Our study also used a mix of individual and group interview methods making group think a potential bias where an individual’s perspective may have skewed the opinions of others participating in the interview. However, the size and scope of our study spanning all regions of the country helped to minimize these biases. Lastly, we did not directly interview patients, limiting our characterization of the patient perspective and the individual patient-level barriers to cervical cancer. However, our study’s main strength lies in the vast breadth of patient advocates from every sector and region of the healthcare pipeline who provided key insights into barriers patients face and where they see room for improvement.

## Conclusions

Despite no-cost cervical cancer screening in Belize, complex barriers still exist. This study provided important insights about the needs of the scalability and success of the cervical cancer screening program. Addressing overloaded provider responsibilities, funding for staff training and facilities, and protocols for introducing new implementation are potential areas for system strengthening. Careful consideration of these barriers when implementing new strategies to control cervical cancer morbidity and mortality (e.g., HPV DNA testing) will assure the effectiveness and sustainability of these interventions.

## Supplementary Information

Below is the link to the electronic supplementary material.

Supplementary file1 (PDF 56 KB)

## Data Availability

The datasets generated during and/or analyzed during the current study are not publicly available due to agreements made with the Ministry of Health Belize to protect the confidentiality of the interviewees.
