# Supplementary material for: Barriers and facilitators to effective cervical cancer screening in Belize: a qualitative analysis
Source: Cancer Causes Control. 2023 May 11;34(8):647–56. doi: 10.1007/s10552-023-01703-0 (PMC10267283; doi:10.1007/s10552-023-01703-0)
Supplement: Supplementary file 1 — Supplementary file1 (PDF 56 KB) [file 10552_2023_1703_MOESM1_ESM.pdf]

Semi-Structured Interview Questions:

1. General
    a. **What is the current situation regarding cervical cancer in Belize?**
        i. Is cervical cancer a problem in Belize?
        ii. Is cervical cancer a problem in your community?
        iii. Please describe the role of your facility/self in cervical cancer prevention and treatment.
2. Demographics
    a. **What are the demographics of the community you serve?**
        i. What is the predominant ethnicity in the community you serve?
        ii. *What are the values of the community you serve as it relates to sexual health and vaccination?*
3. Primary Prevention
    a. **How does your facility serve the HPV vaccination effort?**
        i. What are your thoughts on the HPV Vaccine?
        ii. What are the challenges of the HPV vaccination effort?
        iii. Has the HPV vaccination effort been successful in your community?
4. Secondary Prevention
    a. **How does your facility serve the cervical cancer screening effort?**
        i. What screening services does your facility/self provide?
        ii. What are the challenges of cervical cancer screening in Belize and in your community?
        iii. What other tools are you aware of that could be used for cervical cancer screening? [Follow-up and ask about HPV DNA Testing]
        iv. Have the screening efforts [VIA and/or Pap and/or other] been successful in your community?
        v. *Do patients need to pay for screening services?*
5. Monitoring and Evaluation
    a. **What systems do you/your facility have in place for collecting patient/service data?**
    b. **What systems do you/your facility have in place for quality assurance?**
        i. Do you believe you have too much, too little or a good amount of work to complete as it relates to tracking patient/service data?
        ii. *Does your facility have a cancer registry?*
            1. Would a cancer registry be a feasible implementation?
        iii. *What is the highest fidelity tool for tracking patient information?*
        iv. What are the challenges with tracking patient/service data?
6. <u>Comments on Capacity *late iteration*</u>
    a. *What is the status of the human resources as it relates to your role/facility?*
    b. *What is the status of the infrastructure as it relates to your role/facility?*
    c. *What is the status of funding for your services at your facility?*
    d. *What is the status of policy implementation and development at your facility?*
